# Geopolymers and Fiber-Reinforced Concrete Composites in Civil Engineering

**DOI:** 10.3390/polym13132099

**Published:** 2021-06-25

**Authors:** Aamir Mahmood, Muhammad Tayyab Noman, Miroslava Pechočiaková, Nesrine Amor, Michal Petrů, Mohamed Abdelkader, Jiří Militký, Sebnem Sozcu, Syed Zameer Ul Hassan

**Affiliations:** 1 Department of Material Engineering, Faculty of Textile Engineering, Technical University of Liberec, Studentská 1402/2, 46117 Liberec, Czech Republic; aamir.mahmood@tul.cz (A.M.); miroslava.pechociakova@tul.cz (M.P.); jiri.militky@tul.cz (J.M.); sebnem.sozcu@tul.cz (S.S.); 2 Department of Machinery Construction, Institute for Nanomaterials, Advanced Technologies and Innovation (CXI), Technical University of Liberec, Studentská 1402/2, 46117 Liberec, Czech Republic; nesrine.amor@tul.cz (N.A.); michal.petru@tul.cz (M.P.); 3 Department of Advanced Materials, Institute for Nanomaterials, Advanced Technologies and Innovation (CXI), Technical University of Liberec, Studentská 1402/2, 46117 Liberec, Czech Republic; mohamed.fawzy@mena.vt.edu; 4 Department of Mechanical and Materials Engineering, Vilnius Gediminas Technical University, Sauletekio al. 11, 10221 Vilnius, Lithuania; 5 Department of Nanoengineering, Center for Physical Sciences and Technology (FTMC), Savanoriu Ave. 231, 02300 Vilnius, Lithuania; 6 Department of Textile Engineering, Balochistan University of Information Technology, Engineering and Management Sciences, Quetta 87300, Pakistan; syed.zameer@buitms.edu.pk

**Keywords:** jute, basalt, glass, geopolymers, composites, concrete

## Abstract

This paper discusses the influence of fiber reinforcement on the properties of geopolymer concrete composites, based on fly ash, ground granulated blast furnace slag and metakaolin. Traditional concrete composites are brittle in nature due to low tensile strength. The inclusion of fibrous material alters brittle behavior of concrete along with a significant improvement in mechanical properties i.e., toughness, strain and flexural strength. Ordinary Portland cement (OPC) is mainly used as a binding agent in concrete composites. However, current environmental awareness promotes the use of alternative binders i.e., geopolymers, to replace OPC because in OPC production, significant quantity of CO_2_ is released that creates environmental pollution. Geopolymer concrete composites have been characterized using a wide range of analytical tools including scanning electron microscopy (SEM) and elemental detection X-ray spectroscopy (EDX). Insight into the physicochemical behavior of geopolymers, their constituents and reinforcement with natural polymeric fibers for the making of concrete composites has been gained. Focus has been given to the use of sisal, jute, basalt and glass fibers.

## 1. Introduction

**Figure 1:**
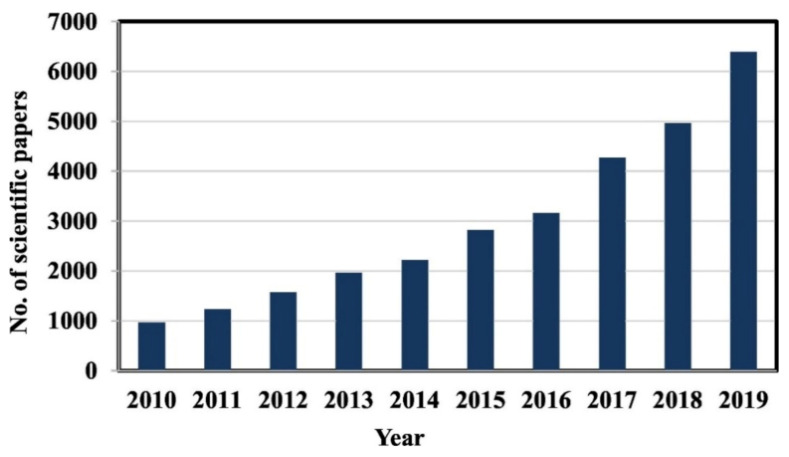
A graphical representation of data published on geopolymers from 2010 to 2020 based on its economical and energy consumption benefits. Reprinted with permission from Ref. [2]. Copyright 2021, with permission from Elsevier.

The modern world is facing many challenges due to a day-by-day deterioration in the natural environment and the depletion of natural resources. New techniques and materials are being sought to combat this situation. The construction industry has been an important contribution to environmental deterioration. The last few decades have witnessed the use of geopolymeric materials in the construction industry in an attempt to address certain environmental and economic concerns. Rapid industrialization, development and innovations in almost every part of the world have brought about an alarming day-by-day deterioration in the atmospheric environment. With an exponential increase in human population, the demand for major construction has increased to fulfil the human necessity for shelter. This can be considered a threat to the depletion of natural resources. Concrete is one of the major materials utilized in the construction industry and it is estimated that the production of concrete is responsible for 5–8% of total CO_2_ emissions in the world. Most, 95%, CO_2_ emissions are due to the fabrication of cement, which is the main constituent of concrete [1]. This reflects the need for the development of more economical and environment friendly materials with lower CO_2_ emissions. In this regard, efforts have been made to find alternatives that can be used with lower pollution and less energy consumption. Various methods for reducing CO_2_ emissions during the fabrication of cement have been examined previously [2]. These methods include the use of alternative fuel [3], improving energy efficiency inside the cement kiln [4], modifying cement with optimum percentage of nanoparticles [5] and creating alternative cement like geopolymers [6]. Among these options, geopolymers with environmentally friendly characteristics are receiving more and more attention from the scientific community and are new entrants in the field of construction materials. The fabrication of geopolymers is cost-effective and requires less energy consumption in comparison with the formulation of other alternatives available. In addition, the inner portion of industrial waste such as fly ash adds many advantages and causes geopolymers to be a completely new green material for use in the construction industry [7,8,9]. Some studies indicate that the use of geopolymers can reduce greenhouse gas emissions from 44% to 64% in comparison to that for the production of ordinary Portland cement (OPC) [10,11,12]. If careful design of geopolymer cement is carried out, its production will require less energy and may reduce CO_2_ emission by 80% compared to those from OPC production [13]. Geopolymers have performance characteristics similar to those of OPC and hence are viewed as one of the most likely alternatives to OPC. OPC can be replaced with geopolymers, which serve as a binding phase and at the same time, can enhance mechanical properties of concrete [14]. The situation described above has motivated scientists from all over the world to focus on the development of geopolymers. Figure 1 shows an increasing interest on geopolymer development due to reduced energy consumption, lower CO_2_ emission, and a high reduction in cost compared to the generation of traditional materials.

## 2. Geopolymers

**Figure 2:**
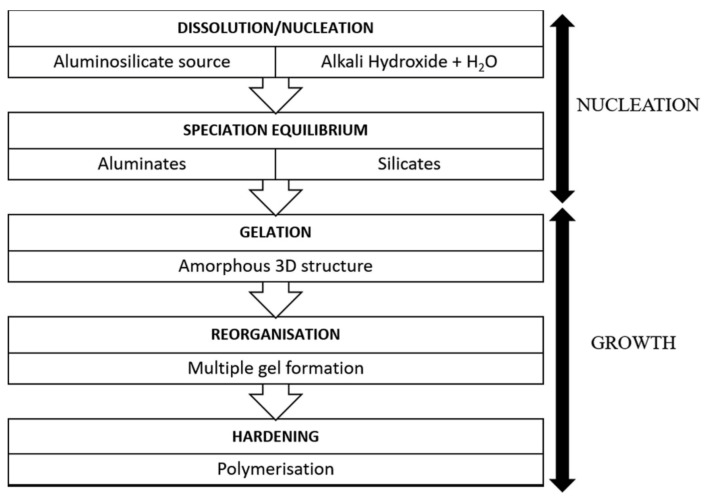
A general flow of geopolymerization process. Reprinted with permission from Ref. [21]. Copyright 2020, with permission from Elsevier.

**Figure 3:**
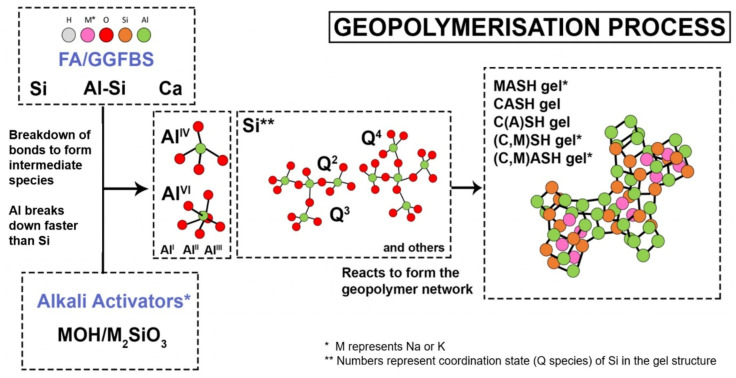
A chemical view of the geopolymerization reaction mechanism of aluminosilicate (breakdown of bonds and formation of intermediate species) dissolution in the activator solution. Reprinted with permission from Ref. [22]. Copyright 2021, with permission from MDPI.

Geopolymers are used in many fields ranging from aeronautics and civil engineering to the plastics industry [15,16,17,18]. The synthesis of geopolymers is based on a chemical reaction between solid aluminosilicate compounds (Si_2_O_5_, Al_2_O_2_) and a highly concentrated alkali hydroxide or polysilicate solution [19]. Kaolin and metakaolin are the examples of naturally occurring aluminosilicate compounds whereas industrial wastes i.e., fly ash and blast furnace slag, are considered to be industrial sources. The dissolution of the aluminosilicate compounds by hydrolysis forms silicate and aluminate due to the presence of alkaline activators. After dissolution, a reaction with silicates takes place promoted by the presence of activators solution. At a higher pH, the amorphous aluminosilicates are quickly dissolved and form a highly saturated aluminosilicate solution that further produces a gel like structure. Large networks are formed by oligomers in an aqueous media due to condensation. This gel-like structure is bi-phasic due to the water and aluminosilicate binder. The conversion time from a supersaturated aluminosilicate solution to gel depends on the synthesis conditions, materials composition and concentration of activators solution. After the gelation process, the system continues rearranging as the linkages of the gel network are enhanced, resulting in the three-dimensional (3D) aluminosilicate network named geopolymers [20]. A general flow diagram of the synthesis of geopolymers is illustrated in Figure 2 [21]. However, a chemical view of geopolymerization process shows that Al-O/Si-O bonds are broken in the solid alumina/silica rich binder material during the hydrolysis by hydroxyl initiator [OH]¯ and produces a tetrahedral aluminate and silicate intermediate species i.e., [Al(OH)4]¯ and [Si(OH)4]^4−^. Water condenses during gelation and an overall shrinkage occurs in the structure. Therefore, the system reorganizes and rearranges itself into a 3D network as explained in Figure 3 [22].

**Figure 4:**
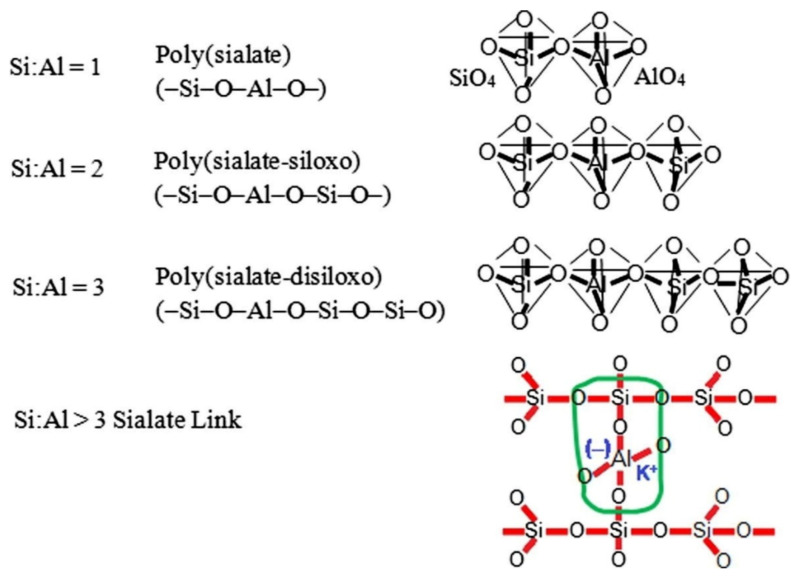
Siloxo Si-O units based on different geopolymeric systems. Reprinted with permission from Ref. [24]. Copyright 2016, with permission from Elsevier.

Geopolymers are categorized on Si/Al ratios, and the three basic geopolymeric structures based on Si/Al ratios are poly (sialate), poly (sialate-siloxo) and poly (sialate-disiloxo) as illustrated in Figure 4 [23,24]. Sialate is the common name for silicon-oxo-aluminate and it ranges from amorphous to semi-crystalline in nature. Therefore, raw materials that contain rich silica and alumina content can be used for the production of geopolymers.

**Figure 5:**
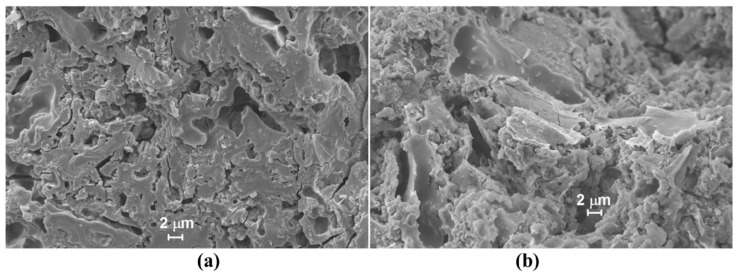
SEM micrographs of illustration of (**a**) geopolymers sample immersed in water exhibited glass-like microstructural surface and (**b**) geopolymers sample after acid exposure showed debris-like colloidal particles (could be the precipitates of silica gel) on the surface. Reprinted with permission from Ref. [30]. Copyright 2018, with permission from Elsevier.

In general, geopolymers are defined as ceramic-like materials formed at room temperature. They are inorganic in nature and contain a aluminosilicate polymer as a building block. Geopolymers can be made from both industrial and agricultural wastes like fly ash, metakaolin, rice husk ash and wheat straw ash [25,26]. Geopolymers are prepared by mixing alkaline activators (mainly CaO) and water along with aluminosilicate-based sources. Alkali metal silicates, hydroxides or even carbonates have been used for making an alkaline solution for the activation. However, some researchers also used NaOH or KOH in this regard [27,28,29]. During the activation process, SiO_4_ and AlO_4_ species were obtained as the solid aluminosilicate oxide dissolution took place and, as a result of the polycondensation reaction, an amorphous three-dimensional geopolymeric system formed. However, the inclusion of acids involves the deterioration of Si-O-Si bonds during geopolymerization or may leave an Al depletion silica system that depends on the distribution of Si and Al in the framework. Figure 5 shows the SEM images of water-immersed geopolymer samples and acid-immersed samples after 70 days of immersion [30].

Geopolymer cement is reported to attain rapid hardening, and after 4 h of manufacturing at 20 °C, it achieves a compressive strength in the range of 20 MPa whereas after 28 days, it gains compressive strength from 70 to 100 MPa. Heat-treated, low-calcium content, fly-ash-based geopolymer concretes have excellent compressive strength and do not suffer from drying shrinkage and low creep [31]. Geopolymers exhibit better chemical resistance to sulphates than OPC. For low-cost ceramic materials at ambient temperatures and for pre-cost applications, drying and hardening of geopolymers after the initial setting is very important especially in applications where shrinkage is not desirable [32]. It is important to define the exact chemical nature of geopolymeric gel, which is further responsible for determining the mechanical properties of geopolymers. It is also important to note that the nanostructure and molecular nature within the gel determine the thermal and chemical stability of the binder. In this regard, correct selection of raw materials and mixing design are essential factors in achieving the enhanced properties of geopolymers for specific purposes. Geopolymers are reported to be fire resistant because of their inorganic framework and show high thermal stability in comparison with OPC [20]. It is also reported that geopolymers have excellent mechanical properties in comparison to OPC [33]. Density, compressive strength, flexural strength and modulus of elasticity are the important mechanical properties of geopolymers for further use in structural applications. Table 1 summarizes the detail of the constituents of geopolymers, their role on the properties of fiber-reinforced composites and the experimental conditions, etc.

### 2.1. Factors Affecting on Geopolymers

Many factors that affect the compressive strength, flexural strength and other mechanical properties of geopolymers have been reported by different researchers [22,38,39,40,41,42]. These factor, such as different calcium-containing raw materials [21,43], ionic additives, curing procedures and post-curing chemical treatment, have been considered important for final properties [44,45,46]. An amorphous structure of geopolymers is better for realizing anticipated mechanical strength. It is important to note that the properties of geopolymers greatly depend on the SiO_2_/Al_2_O_3_ ratio, NaOH/Al_2_O_3_ or SiO_2_/KOH ratio and the liquid–solid ratio. Research works investigating the influence of the C-S-H phase on the geopolymerization of aluminosilicates with a focus of its role on early-age strength have been made previously [47,48,49]. Phair and Deventer investigated the C-S-H phases and demonstrated C-S-H phases at different pH levels. According to them, the presence of C-S-H at pH 12 did not improve the compressive strength significantly as compared to pH 14 [50].

The effect of admixtures on geopolymers is another important factor that alters the overall properties. It has been noted that sucrose and citric acid, as admixtures that perform the role of retarder in OPC, have dissimilar mechanisms in fly-ash-based geopolymers [51]. Commercial superplasticizers such as naphthalene and polycarboxylate based superplasticizers were also investigated. It is reported that a naphthalene-based superplasticizer is effective when a single activator is used, rendering a 136% increase in relative slump without disturbing the compressive strength. When a multi-compound activator is used, a modified polycarboxylate-based superplasticizer is more effective [52,53]. The retarding effect of a polycarboxylate-based superplasticizer in a fly ash/slag blended system is reported in the literature along with a significant improvement in workability compared to a naphthalene-based superplasticizer.

Many researchers have conducted studies on the properties of geopolymer pastes based on various curing conditions. For achieving complete geopolymerization, researchers reported curing of samples at temperatures between 40 °C and 85 °C. Palomo et al. reported that the alkali-activated fly ash, when cured at a temperature 85 °C for 24 h, gave significant compressive strength of the geopolymer as compared to the same composition at 65 °C [54]. However, no significant increase in compressive strength was noted with curing time extended beyond 24 h. Heah et al. showed that metakaolin-based geopolymers cured at higher temperatures result in an increase of strength after 1 to 3 days. They also discovered that samples cured at a higher temperature for a longer time period result in sample failure. This failure was described by the thermolysis of the -Si-O-Al-O- bond [55]. According to Rovnanik, a metakaolin-based geopolymer cured at a higher temperature (40–80 °C) showed deterioration of mechanical properties when compared with the results obtained for slightly decreased temperature. In order to achieve better mechanical and durability properties of geopolymers, suitable curing is a must [56]. Previous research has shown that the mechanical properties of geopolymer are strongly influenced by its Si/Al ratio. Additionally, the liquid/solid ratio, which is linked to the water content in the reactive mixture, has an effect on the geopolymers formulation. Zuhua et al. demonstrated that an increased liquid/solid ratio supported the transfer of ions and thus boosted the dissolution of the aluminosilicate source. On the other hand, this increase slowed the polycondensation reactions and resulted in an increased porosity rate, thus leading to poor mechanical performance [57].

### 2.2. Cementitious Materials for Geopolymers

Commonly cementitious materials are created by alkaline activation either based on Si and Ca or based on Si and Al. In recent times, efforts have been made to replace OPC with other cementitious materials containing Si and Al. Various activated natural materials and industrial by-products are being used to produce alkaline-activated binders for further use in developing cementless mortar and concrete. Supplementary cementitious materials for geopolymers have less environmental impacts compared to OPC. Research work on geopolymers has indicated that the inclusion of raw materials such as fly ash and slag in geopolymers fabrication is gaining interest worldwide, and these industrial waste materials are responsible for deciding the final properties of geopolymers [58,59]. In this regard, most of the researchers have focused on employing fly ash/blast furnace slag for geopolymers systems. Microstructure, physical, mechanical, chemical and thermal properties of geopolymers, in contrast to their macroscopic characteristics, mainly depend on the raw materials. Materials that are rich in aluminum and silicone can be used in the fabrication of geopolymers. These materials include fly ash, slag, waste glass and some pure Al-Si minerals and clays (kaolinite and metakaolinite). Among them, fly ash and slag are the most widely used ones.

#### 2.2.1. Fly Ash

**Figure 6:**
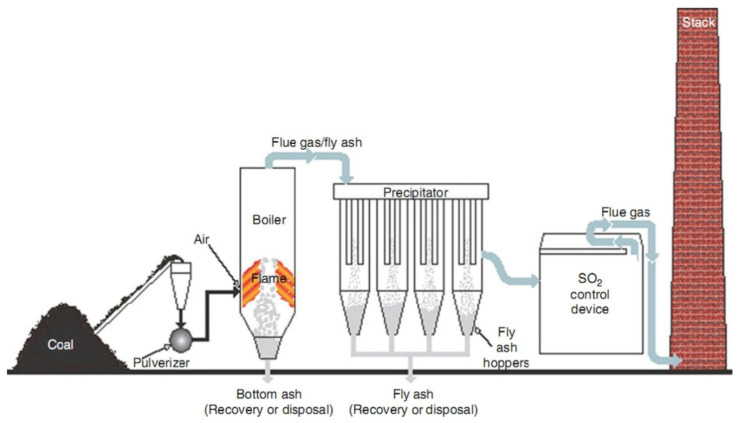
A schematic depiction of a clean production process of fly ash from coal. Reprinted with permission from Ref. [41]. Copyright 2021, with permission from Elsevier.

Fly ash is a residue generated in coal-fired power plants for electricity generation and is viewed as solid waste material [60]. Fly ash is produced at an amount of about 800 million tons annually, worldwide. China is the largest country in fly ash production followed by India, USA and EU [61]. It has been reported that the fly ash utilization rate is estimated as 50% for the USA, more than 90% for EU, 60% for India and 67% for China. Coal fly ash usually consists of coarse bottom ash and fine fly ash, of which coarse bottom ash is about 5–15% by weight and fine fly ash is about 85–95% by weight of total coal ash generated. Bottom ash accumulates on the bottom of the boiler by air flow whereas the fly ash is caught from the flue gas and collected by electrostatic or mechanical precipitation. Fly ash particles are mainly spherical and comprise solid spheres, cenospheres, irregular shaped waste and porous unburnt carbon [62]. Fly ash is considered to be a pozzolan-like material at room temperature when mixed with water and calcium hydroxide form cementitious products. The color of fly ash is generally grey, and the amount of unburned coal in the ash is responsible for defining its color from dark to dull to black, with fine, powdery particles mostly spherical in shape. These particles are either solid or hollow and predominantly amorphous [63]. The type of coal used at power plants significantly influences the physical and chemical characteristics of fly ash [64]. Generally, fly ash contains quartz, hematite, mullite and amorphous particles. Chemical composition of fly ash is greatly influenced by factors such as the type of coal used for burning and conditions under which this process takes place, as well as the removal effectiveness of the air-pollution-control device [65]. A complete production process of fly ash (from coal and after pulverization, boiling and precipitation) is thoroughly explained in Figure 6 [41].

Fly ash can be classified based on chemical and mineralogical composition into two types, that is, class C and class F, depending on the coal used for combustion. Class C fly ash is obtained during the combustion of lignite and sub-bituminous coals and contains SiO_2_, Al_2_O_3_ and Fe_2_O_3_, together less than 50%, whereas CaO ranges between 20% and 30%. On the contrary, class F fly ash is obtained when anthracite and bituminous coals are burned, and contains more than 70% of SiO_2_, Al_2_O_3_ and Fe_2_O_3_ with CaO content less than 5% [66]. Generally bituminous coal fly ash particle size is less than 0.075 mm and is normally similar to silt particle size, whereas sub-bituminous coal fly ash is also similar to that of silt particles, but is a little coarser compared to bituminous coal fly ash. The specific gravity of fly ash is found to be between 2.1 and 3 and specific surface area between 170 and 1000 m^2^/kg. The bulk density of fly ash is in the range of 0.54–0.86 g/cm^3^. Properties of fly ash of classes C and F differ significantly and hence both classes have different uses. Class C fly ash is characterized as high-calcium fly ash and can be considered as a cementitious material if CaO content is higher than 20%. High-calcium fly ash with CaO between 10% and 20% is said to be a cementitious and pozzolanic material [67]. Past years have witnessed increasing research on fly ash and its industrial utilization. Fly ash has been investigated mainly in areas such as cement fabrication, ceramics, paints, plastics, agriculture and construction industry [68]. Low-calcium fly ash represents the properties of normal pozzolan (a material with silicate glass and modified with aluminum and iron). Pozzolanic activity to form strength-developing products with low-calcium fly ash takes place when it interacts with Ca(OH)_2_. For this purpose, low-calcium fly ash is used in combination with OPC to produce Ca(OH)_2_ during the hydration process. On the other hand, the suitability of high-calcium fly ash in concrete is viewed with doubts. This can be explained by high free CaO and sulphur content in such a fly ash chemical composition that can disturb concrete volume stability and durability. Yet, high-calcium fly ash can cause early strength development in concrete and, if proportioned accurately, can increase the quality of concrete.

**Figure 7:**
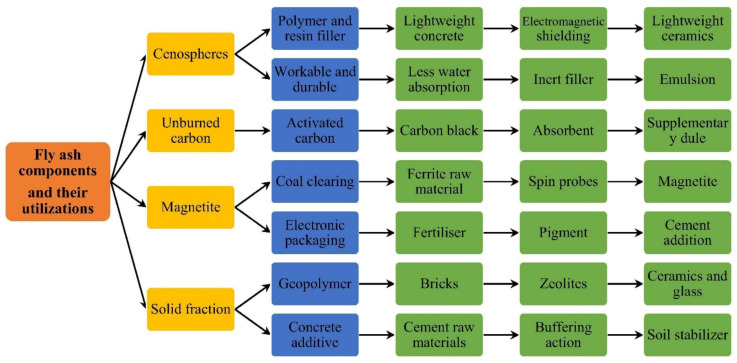
The utilization of fly ash and its components into different real-life applications. Reprinted with permission from Ref. [41]. Copyright 2021, with permission from Elsevier.

Fly ash as a replacement for cement in concrete is limited to 15–20% by mass of the total cementitious material [69]. However, Malhotra reported that more than 50% of fly ash replacement in concrete can be used, subject to the fact that acceptable material responses, such as strength, durability, permeability and shrinkage, are ensured [70]. Fly ash increases the workability and lessens the bleeding of fresh concrete. It also increases the durability properties of concrete and, if designed properly, exhibits enhanced strength and low permeability. Concretes with partial replacement of OPC with fly ash show considerable increase in workability compared to OPC concrete. Fly ash containing concretes show increased workability with increasing levels of fly ash replacement thus reducing the water demand for the system [71]. Fly ash can be used for controlling sulphate attack and both classes of fly ash behave differently in such a corrosive environment. High-calcium fly ash contains considerable amounts of soluble calcium, aluminum and sulphur-bearing minerals as well as a substantial amount of calcium aluminate glass, which can release calcium and aluminum into the solution slowly. It increases the pH of the solution when it comes in contact with water and the expansion of cement occurs, thus leading to cracking. Class C fly ash increases the exposure to sulphate attack as it is rich in lime and hydrates independently. On the other hand, class F fly ash hinders the sulphate attack by hampering the formation of alumino silicate hydrate compounds. It has been reported that low-calcium fly ash concrete is more resistant compared to concrete with high-calcium fly ash [72]. A flow of fly ash and its components in the utilization of real-life applications in various industries is presented in Figure 7 [41].

#### 2.2.2. Ground-Granulated Blast Furnace Slag (GGBFS)

**Figure 8:**
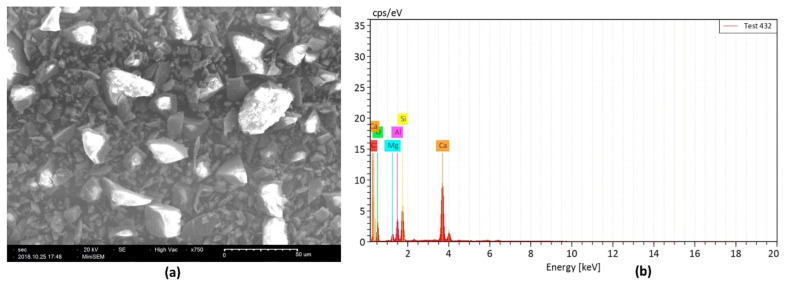
Results of ground granulated basalt furnace slag from (**a**) SEM analysis shows different grades of denser particles and (**b**) EDX analysis shows higher silicon content than OPC. Reprinted with permission from Ref. [74]. Copyright 2021, with permission from Elsevier.

Ground-granulated blast furnace slag (GGBFS) is among the other by-products during the production of iron and steel. During the process of iron and steel production in blast furnaces, quick removal of slag on the top of molten iron is carried out and then grounded to obtain GGBFS. It is a powder-like material and white in color. Physical properties of GGBFS depend on the cooling process and chemical properties depend on the selection of raw materials for iron production. Its fineness specific surface area is between 300 m^2^/kg and 500 m^2^/kg with specific gravity ranging from 2.4 to 3.0 [73]. GGBFS is pozzolanic in nature, and for decades, has been used as cementitious component for making cement/concrete composites. The effectiveness of GGBFS in cementitious composites depends on many factors, such as chemical composition, fineness and hydraulic reactivity. SEM and EDX analysis for GGBFS explains that GGBFS contains denser particles and those particles have different size grades that work as pore filtration as well as higher silicon contents than OPC, as illustrated in Figure 8 [74].

GGBFS is non-metallic in nature and basically contains oxides of calcium, silica, alumina and magnesia along with some other oxides in small quantities. Both glassy and crystalline phases are present in GGBFS with glass content in the range of 85–90% [75]. The amount of glassy phase in GGBFS is very much affected by the cooling process. Slow cooling produces about 50% to 70% of the crystalline phase in GGBFS, which in turn reduces the hydraulic activity. On the other hand, quick cooling normally develops smaller and uniform particle sizes in GGBFS and contributes to advanced hydraulic activity. A number of formulas have been developed to predict the hydraulic activity of GGBFS. These formulas do not represent the exact strength performance of GGBFS because the hydration reactions that take place are very complex in nature and these formulas do not indicate them exactly [76].

Chemical composition is vital for achieving good hydraulic activity of GGBFS. GGBFS are classified based on their basicity index. A CaO/SiO_2_ ratio more than 1.0 is one such basicity index described in the literature. Basicity defines the hydraulic activity of the slag, and if the slag is more basic, its hydraulic activity in the presence of some alkaline activator is better. With constant basicity, an increase in Al_2_O_3_ content increases the strength, whereas variations in MgO content reaching 8–10% can insignificantly affect the strength development while exceeding 10% can affect it negatively. Furthermore, increasing the amount of CaO increases the hydraulic activity of GGBFS whereas it decreases with an increasing content of SiO_2_. In order to assure high alkalinity, without which GGBFS is hydraulically inactive, European Standard 197-1 suggests a ratio of the mass of CaO and MgO together to the mass of SiO_2_ ratio greater than 1.0. It has been observed that initial hydration is considerably slower when GGBFS is hydrated with water only. To overcome this, OPC, alkalis or lime are used as activators to accelerate the hydration process [77]. Like other cementitious materials, surface area is used to determine the reactivity of GGBFS. Increased fineness gives better strength development. Setting time, shrinkage and economic considerations are the factors that limit fineness practically. Fineness of GGBFS affects the reactivity of slag in concrete, early strength development and water requirement. On the other hand, it is dependent on energy saving and economic considerations. It has been reported that the fineness of GGBFS should be two to three times greater than that of OPC in order to have advantages over slag in certain engineering properties like bleeding, setting time, high strength and excellent durability.

A number of research works have been carried out to study the workability of concrete and mortar with the inclusion and replacement of GGBFS. It has been reported that 60% replacement of OPC with GGBFS in concrete improved its workability. Generally, replacing OPC with an increasing percent of GGBFS results in prolonged setting times of concrete whereas replacement equal to or more than 40% has an extremely retarding effect. The use of GGBFS for applications where early-age strength is required is not recommended. Concrete with the inclusion of GGBFS under a normal temperature curing condition attains considerably slow strength development as compared to OPC concrete. However, the early strength development of GGBFS included considerable concrete increases at higher early-age temperatures [78]. In a study carried out by Oner and Akyuz, it was observed that the compressive strength of GGBFS concrete increased with an increase in GGBFS content and an optimum level for the efficient use of GGBFS content was around 55–59%. Flexural strength of concrete is prone to micro-cracking, and the inclusion of GGBFS can improve its flexural strength at later stages [79]. The influence of GGBFS on the flexural strength of concrete showed that concrete containing 60% of GGBFS was higher than the flexural strength of the same concrete without GGBFS whereas a noticeable decrease was observed with 80% replacement.

#### 2.2.3. Metakaolin

**Figure 9:**
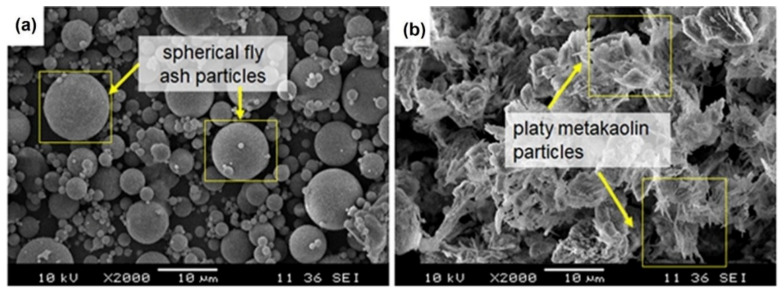
SEM images of (**a**) fly ash and (**b**) metakaolin. Reprinted with permission from Ref. [83]. Copyright 2021, with permission from Elsevier.

Less energy-intensive processed materials and wastes with pozzolanic behavior have been using by mankind for thousands of years. One such example in recent times is metakaolin [80]. Metakaolin belongs to the calcined clay family and is obtained from the calcination of kaolin clay. The use of kaolin as an industrial mineral in various industries largely depends on its mineralogical composition, geological conditions under which it formed and its physical and chemical properties. Kaolin is found as sedimentary, residual or hydrothermal and has unique properties in every of these cases, and therefore is subject to proper testing and evaluation for a specific use. Kaolin deposits are found in southwestern England, Georgia and South Carolina in the USA as well as in the lower Amazon of Brazil. which are known as the most utilized ones. Kaolinite crystals that cover most of the kaolin deposits are pseudo-hexagonal along with plates, some larger books and vermicular stacks [81]. Metakaolin is a highly reactive pozzolana type and needs to be processed like cement in burning kilns at temperatures around 700–900 °C and mainly contains silica and alumina. Metakaolin is produced under these temperatures, when kaolin-clay rich in kaolinite undergoes thermal activation and by dehydroxylation, which leads to breaking down or partial breakdown of the structure resulting in a transition phase with high reactivity [82]. The morphologies of fly ash and metakaolin particles are observed by SEM analysis and illustrated in Figure 9 [83]. It is observed that fly ash particles are quasi-spherical and smooth with a variation in sizes. However, metakaolin particles are mostly irregular in terms of shape. The quasi-spherical-shaped particles of fly ash are more suitable for lower water demand than metakaolin particles.

Metakaolin in the presence of water reacts chemically with Ca(OH)_2_ produced by cement hydration and this generates additional gel containing secondary calcium-silicate-hydrate (C-S-H) along with crystalline products including aluminate hydrates and aluminosilicate hydrates. The hydration reaction is associated with the metakaolin reactivity level and is influenced by the processing conditions and purity of the clay type used [84]. Metakaolin is an off-white powder type of aluminosilicate material and has porous, angular-shaped, platy, smaller particle size of about 1–2 µm with a surface area in the range of 10,000–29,000 m^2^/kg. Its specific gravity is in the range of 2.20–2.60 whereas the bulk density of metakaolin has been reported in the range of 300–400 kg/m^3^ [85,86,87]. The chemical composition of metakaolin varies according to the type of kaolin used. The main components of metakaolin are silicon dioxide and alumina oxide with other components as well including ferric oxide, calcium oxide and potassium oxide. As per ASTM standard, raw or calcined natural pozzolan is characterized as class N with SiO_2_, Al_2_O_3_ and Fe_2_O_3_, together equal or greater than 70%, SO_3_ less than or equal to 4%, limiting oxygen index (LOI) content less than or equal to 10% and moisture content less than or equal to 3%.

Metakaolin as a pozzolanic addition has attracted much interest in recent years. The main interest regarding using metakaolin in mortar or concrete is the removal of C-H produced by the hydration of cement responsible for poor durability. Furthermore, C-H removal guarantees improved strength and has a key influence on resistance to sulphate attack and alkali silica reaction. Purity of metakaolin, OPC composition, water/binder ratio and curing conditions need to be addressed for the metakaolin content in a system for complete elimination of C-H [88]. Concretes with partial replacement of OPC with metakaolin shows a considerable reduction in workability and that the degree of workability ranges from low to very low with the increase in replacement, thus increasing the water demand of concrete. Despite a lower degree of workability, metakaolin concretes can be compacted well without any difficulty. An increase in compressive strength is caused by the filling effect, speeding up of cement hydration and pozzolanic reactivity of metakaolin in concrete containing metakaolin. Previous research showed that metakaolin-based concrete with the partial replacement of OPC with metakaolin ranging 5–15% achieved a cube compressive strength of 92–104 MPa at 28 days, whereas for the same samples, static and dynamic moduli of elasticity were observed as 45.73–46.26 and 51.78–52.86 GPa, respectively. Generally, metakaolin geopolymers set and harden within 24 h, with the short setting time of 4 h. With high Al_2_O_3_ content, metakaolin geopolymers’ setting time is short but it weakens the strength with a low content of SiO_2_.

**Figure 10:**
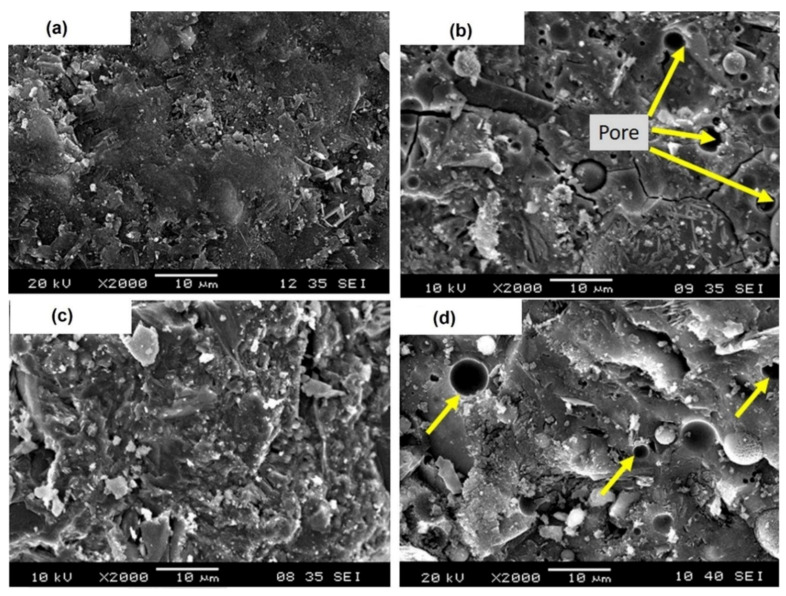
The morphologies of fly ash and metakaolin blended geopolymers composites with (**a**) 1.0 wt% monoaluminium phosphate, (**b**) 3.0 wt.% monoaluminium phosphate, (**c**) 1.0 wt.% aluminum dihydrogen triphosphate and (**d**) 3.0 wt.% aluminum dihydrogen triphosphate. Reprinted with permission from Ref. [83]. Copyright 2021, with permission from Elsevier.

Metakaolin as a partial replacement in concrete acts as a filler and significantly improves early strength and increases long-term strength of concrete. Porosity and pore size distribution measurements observed previously show that the early strength development is linked with considerable pore refinement [89]. Th partial replacement of OPC with up to 20% metakaolin causes improvement in the pore structure of mortar paste, thus increasing the compressive strength. It has been observed that the filler effect is immediate and the speeding up of OPC hydration occurs significantly within the first 24 h whereas the maximum pozzolanic effect takes place between 7 and 14 days. Sulphate resistance of mortar increases by replacing cement with at least 30% metakaolin due to the reduction in C-H content in the paste, which reduces the gypsum and ettringite formation. On the other hand, it is also believed that refinement in pore structure hinders the entrance of suphate ions into the system. The inclusion of metakaolin also improves the resistance of concrete against chloride ions. Concrete with 10% metakaolin has been reported to be effective under freezing and thawing [90]. It has been observed that pozzolanic constituent materials like metakaoline affect the fire resistance of concrete. Metakaolin-based concretes show poor fire resistance at elevated temperatures as compared to OPC concretes, and this is because of the compacted micro-structure and poor porosity of the metakaolin-based concretes. Therefore, careful investigation of metakaolin-based concretes should be carried out for elements that are prone to high temperatures. Figure 10 shows the metakaolin and fly ash blended geopolymer composites with different formulations of monoaluminium phosphate and aluminum dihydrogen triphosphate that provided excellent mechanical and physical properties after a 28-day curing process [83]. It is observed that both fly ash and metakaolin particles provide more compact and homogenous microstructure of the composite that leads to higher compressive strength. In simple words, the addition of fly ash and metakaolin particles into the composite matrix increases the bonding between the particles and the composite matrix. Table 2 elucidates the detail of chemical composition and physical properties i.e., specific surface area, specific gravity, bulk density of fly ash produced with different coal types, GGBFS and metakaolin.

### 2.3. Activating Chemical Solutions for Geopolymers

Chemical activation is used to enhance the reactivity process and improve the fresh and or hardened state. Geopolymers are synthesized when aluminosilicate powders are mixed and activated in highly concentrated alkaline medium [91]. A strong alkaline medium is necessary to dissolve the quantity of aluminosilicates. Usually, alkali earth metal hydroxides and carbonates are used for this purpose. Sodium hydroxide is the most widely used one in this regard. In order to activate the aluminosilicates for geopolymerization, alkali hydroxides or carbonates with pH values more than 13 are required. Alkali metal hydroxides can significantly increase the solution viscosity at concentrations of more than 10 M. When sodium hydroxide is used in excess for activation, the formation of white crystalline sodium carbonates is a well-known issue. There are two types of activating mechanism in geopolymers, the liquid-activated geopolymer binder and the powder-activated geopolymer binder. The former relates to the alkali metal hydroxides, carbonates or silica fume and water whereas the latter relates to the mixing of alkali metal hydroxide and silicates in dry form. Liquid-activated geopolymer binders have been used in most of the studies and have some problems, such as high pH and variations in molarity leading to inconsistent performance [92]. As stated above, sodium hydroxide is the most commonly used alkaline medium for geopolymerization. Sodium hydroxide is commonly known as caustic soda and is a white solid inorganic material and is produced by 50% mass saturated in solution with water. Sodium hydroxide produces a huge amount of heat when it reacts with water. Sodium carbonate is an inorganic material white in color comprising sodium, carbon and oxygen elements, often called washing soda. It is a basic salt with a strong alkaline taste. Potassium hydroxide is an inorganic compound comprising potassium, oxygen and hydrogen elements also known as caustic potash. It is soluble in water and exothermic in nature.

Görhan and Kürklü found that fly-ash-based geopolymers prepared with low and high concentrations of sodium hydroxide exhibited poor compressive strength. They used 3, 6 and 9 M sodium hydroxide concentrations for the samples and noticed that samples with the 6 M concentration exhibited higher compressive strength of 22 MPa [93]. High-calcium, fly-ash-based geopolymers at 70%, 80%, 90% and 100% by mass of binders in combination with OPC and activated by sodium silicate and sodium hydroxide (alkali liquid/binder = 0.65 and Na_2_SiO_3_/NaOH = 0.67) cured at 60 °C showed that samples had increased compressive strength [94]. Fly-ash-based geopolymer mortar with sodium hydroxide as an activator showed increased compressive strength as compared to the same fly-ash-based geopolymer with a potassium hydroxide activator. The compressive strength recorded was 65.28 MPa for the sodium-hydroxide-activated, fly-ash-based geopolymer and 28.73 MPa for the potassium-hydroxide-activated, fly-ash-based geopolymer [95]. Zhang et al. carried out a study on a metakaolin-based geopolymer using sodium hydroxide, potassium hydroxide and sodium silicate as alkali activators with 8 M and 12 M, respectively. The highest compressive strength was observed as 40 MPa with 12 M concentration and with Si/Al = 1.9:1 [96]. In recent research, geopolymers with a combination of metakaolin and low-calcium fly ash (five different combinations of metakaolin and fly ash with metakaolin to fly ash mass ratios of 100:0, 80:20, 50:50, 20:80, 0:100) were activated by sodium hydroxide and potassium hydroxide solutions of 6 M separately. Researchers found that samples activated by sodium hydroxide showed improved compressive strength as compared to the samples activated by potassium hydroxide. GGBFS-based geopolymer concrete with sodium hydroxide and sodium silicate as alkali activators showed that the compressive strength of geopolymer concrete cured at room temperature for 28 days had a maximum value of ~44 MPa when 19 M NaOH solution with 50% Na_2_SiO_3_ concentration was used [97].

## 3. Geopolymers and Natural Fiber-Reinforced Composites

**Figure 11:**
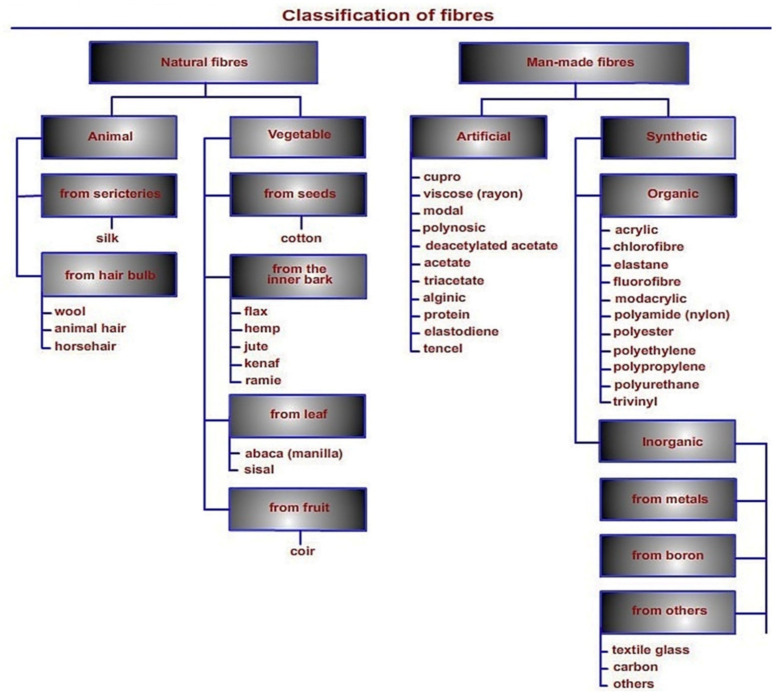
Classification of textile fibers.

Geopolymers exhibit good thermal and durability properties, and at the same time, they are brittle in nature, show poor resistance to tensile and flexural loadings and undergo sudden failure, hence are not suitable for several structural applications [98,99]. To address this issue, research works have been focused on reinforcing geopolymers with synthetic and natural fibers in order to increase their ductility and resistance to tensile stresses. The incorporation of natural fibers in geopolymers gives a feasible solution to counter its initial brittle behavior [100]. Fiber can be defined as a hair-like material that is either a continuous filament or discrete elongated piece similar to thread. Fibers can be broadly divided into natural and man-made ones [101,102]. Figure 11 describes an overview of fiber classification.

In order to increase the flexural strength and energy absorption, fibers can be used as reinforcement in geopolymer composites in the form of threads, filaments, whiskers and nanoparticles. The inclusion of random short fibers in a cementitious medium enhances toughness, ductility and strength by bridging and reducing the cracks [103,104]. Moreover, the addition of fibers in the geopolymer matrix also increases its energy absorption and resistance to deformation. Geopolymers reinforced with any type of fiber show better toughness results in comparison with OPC-based composites. Several factors influence fiber performance in geopolymer composites, including inherent properties of the fiber used, its content, precursors, curing and age of the composites. Yet, the main role for overall mechanical properties is the interface between the fiber and matrix, and with a strong contact interface, high loads can easily be transferred from the matrix to fibers. Most research works concerning fiber-reinforced geopolymers have been done using steel fibers, carbon fibers, glass fibers, polypropylene fibers, polyvinyl alcohol fibers and basalt fibers [105,106,107]. Using cellulosic fibers as reinforcement in recent times has been witnessed as well. In this paper, our focus is on sisal, jute, basalt and glass fiber-reinforced geopolymers composites.

### 3.1. Cellulosic Fiber Reinforced Geopolymer Composites

During the past years, natural cellulosic fibers composites have gained considerable attention due to their low cost and low density and their use as a renewable source [108,109]. Cellulosic fibers have been used as an alternative instead of steel or synthetic fibers within cementitious composites as reinforcement [110]. Cement-based geopolymers reinforced with cellulosic fibers show enhanced toughness, ductility, flexural capacity and crack resistance in comparison to cement-based composites without fiber reinforcement. Fiber reinforcement presents its main benefit during cracking where fibers bridge the matrix cracks and transfer the loads, thus presenting more demanding use of such materials in the construction industry [111,112,113]. However, they have some disadvantages including low durability, efficiency at high fiber content that reduces the workability of a fresh composite, inconsistent material properties and poor interaction with the matrix [114,115]. As mentioned earlier, using cellulosic fibers has the problem of low durability. The use of cellulosic fibers as reinforcement in cement-based materials is limited due to relatively low degradation resistance in alkaline environments. Lignin and hemicellulose phases of natural fibers placed in OPC dissolve, therefore weakening the fiber structure and leading to their degradation in a highly alkaline environment. It has been reported that with an increase in fiber diameter, the corresponding mechanical strength and modulus of fibers decrease. Al-Oraimi and Seibi reported that a small percentage of natural fibers caused an improvement in mechanical properties and impact resistance of concrete, and has the same performance as that of synthetic fiber concrete [116]. Ramakrishna and Sundarajan reported that the addition of fibers increased impact resistance 3–18 times greater than those of without any fibers [117].

#### 3.1.1. Sisal and Its Composite

Sisal fiber is among the most commonly used cellulosic natural fibers. Plant Agave sisalana is the source of sisal fiber. The name sisal fiber comes from Mexico where Maya Indians used it for making ropes, clothes and carpets. Sisal fiber is mainly grown in Brazil, Haiti, East Africa, India, Indonesia and China. The current estimated production of sisal fiber is around 0.30 million tons per year [118,119,120,121]. In every sisal plant, there are 200–250 leaves with 1000–1200 fiber bundles in each leaf comprising 4% fiber, 0.75% cutile, 8% dry matter and 87.25% water. Each leaf of sisal contains three forms of fibers, namely mechanical, ribbon and xylem, of which the mechanical one is the most commercially useful and is extracted from the periphery of the leaf. Sisal fibers can be extracted by either retting or by mechanical means through decorticators. The mechanical method is more suitable for obtaining good-quality fibers extracted with average yield 2–4% (15 kg/8 h). The chemical composition of sisal fiber constitutes 67–78% cellulose, 10–14.2% hemicellulose, 8–11% lignin and 2% waxes [122]. Sisal fibers have low density and relatively high tensile strength. The density of sisal fiber is in the range of 1.03–1.45 g/cm^3^, the tensile strength of sisal fiber is in the range of 347–700 MPa, its Young’s modulus is reported in the range of 15.4–18.79 GPa whereas moisture content is reported as 11%. Elongation at break for sisal fiber was reported to be 2.0–2.5% [123].

**Figure 12:**
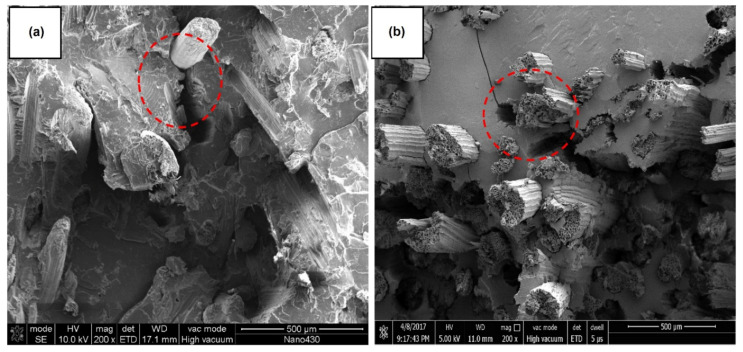
SEM images of (**a**) short sisal fiber-reinforced composites and (**b**) long sisal fiber-reinforced composites. Reprinted with permission from Ref. [125]. Copyright 2021, with permission from MDPI.

**Figure 13:**
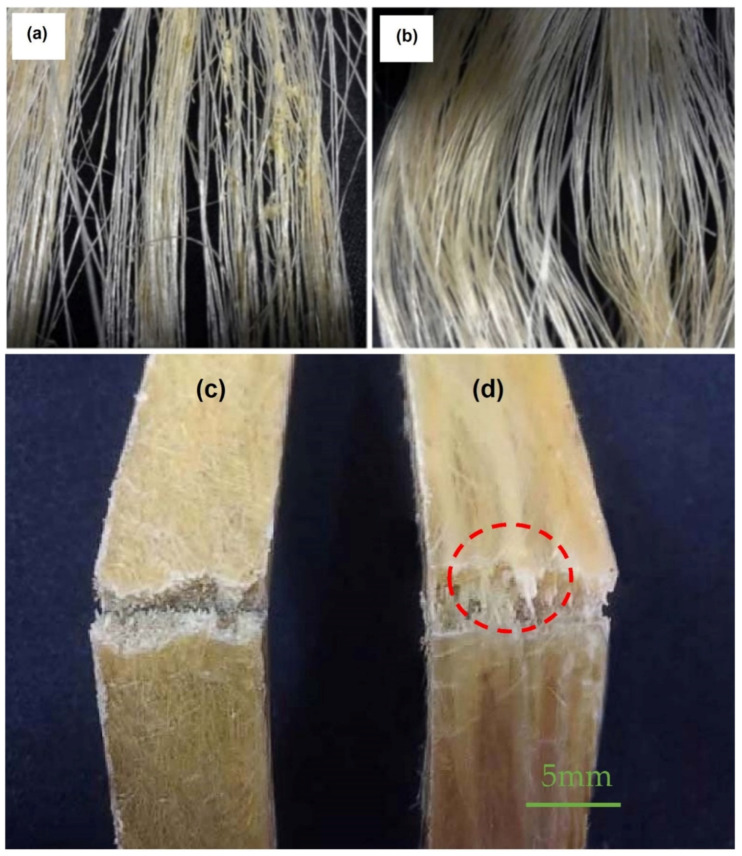
Sisal fibers (**a**) before alkali treatment, (**b**) after alkali treatment, (**c**) flexural strength test for short sisal fibers reinforced composites and (**d**) flexural strength test for long sisal fibers reinforced composites. Reprinted with permission from Ref. [125]. Copyright 2021, with permission from MDPI.

Sisal fiber possesses a higher percentage of cellulose among the different plant-leaves cellulosic fibers and hence is thought to increase tensile properties. Moreover, sisal fibers have higher resistance to water permeability, thus making it attractive for use in construction applications. Sisal-reinforced composites represent higher impact strength with moderate tensile and flexural strengths [124]. In recent years, there has been a continuous effort for the development of sisal-fiber-reinforced composites with high strength in tension and compression. Liang et al. worked with long sisal fibe-reinforced and short sisal fiber-reinforced biocomposites and observed significantly high mechanical properties for long sisal fiber-reinforced composites. The results reveal holes and a fractured and uneven surface of composites for short fibers due to fibers debonding from the matrix during composite destruction. However, the fractured surface was clean, and the polymer matrix had a good wrapping effect on longer sisal fibers. The results of short sisal fiber-reinforced composites and long sisal fiber-reinforced composites after alkali treatment are illustrated in Figure 12 and Figure 13, respectively [125]. It has been reported that reinforcing sisal with thermosets and thermoplastics results in increased mechanical properties. Prasad and Rao prepared composites using sisal fiber as a reinforcement in polyester resin matrix. The results reveal that the tensile strength of sisal/polyester increased by around 52%, tensile modulus increased by 67%, flexural strength increased by around 45% and flexural modulus increased by around 38% [126]. Hashmi et al. prepared the sisal/polypropylene composite and observed that the tensile strength increased by around 36%, flexural strength increased by 25.54% and impact strength increased by 56.52% [127]. In a recent study, Castro et al. investigated mechanical properties of untreated woven sisal fiber-reinforced green high-density polyethylene composites. They produced the composites using woven sisal fibers with a mass percentage proportion of 30:70 (fiber/matrix) and arranging 0°/90° and ±45° stacking sequences and under low-cost manufacturing process based on hot compression molding. Their results showed a 39% increase in tensile strength, a 13% increase in flexural strength, a 35% increase in flexural modulus and a 68% increase in ultimate strain in comparison with traditional polyethylene without sisal fibers [128]. In another study, sisal fiber was reinforced with poly-lactic acid (a biodegradable polymer). Results showed that with sisal fiber inclusion, tensile strength, flexural modulus and impact strength increased by 13.33%, 58.57% and 56.66%, respectively, whereas tensile modulus and flexural strength decreased by 17.24% and 2%, respectively [129]. Savastano et al. used 8% sisal pulp and blast furnace slag as a binder along with OPC in order to prepare composites and observed a maximum flexural strength in the range of 18–20 MPa with an improvement of at least 58% as opposed to that of the composites with fiber inclusion. Using kraft pulps from sisal and banana waste and from Eucalyptus grandis pulp mill residue as fiber reinforcement in cement-based composites showed an optimum performance with a 12% mass fiber content, a flexural strength of around 20 MPa and fracture toughness in the range of 1–1.5 kJ/m^2^ [130].

Baloyi et al. prepared sisal–glass composites by layering methods with varying sisal fiber content treated with NaOH. They found that sisal fiber treated with 20% NaOH placed in nine layers with four layers of glass showed a tensile strength of 57.6 MPa and a flexural strength of 36 MPa [131]. Bahja et al. studied the effect of different treatments on the morphological properties of the sisal fiber and their effect in cement mortar. Their results showed that sisal fiber treatment results in an increased thermal resistance. The addition of sisal fibers by 4% mass of cement decreased the density of mortar and increased its porosity. A reduction in compressive and flexural strengths of the mortar with treated sisal fibers was noticed with satisfactory results for the elastic modulus of mortar [132]. Ren et al. conducted a study on the mechanical properties of sisal fiber-reinforced ultra-high-performance concrete. Samples were prepared using 1, 2 and 3% sisal fibers with lengths of 6, 12 and 18 mm. They noticed that sisal fiber had little effect on compressive strength of ultra-high-performance concrete and reduced the flowability of the concrete as well. Their results revealed that with 2% content of sisal fibers of 18 mm length, the flexural strength and toughness of the ultra-high performance concrete increased by 16.7% and 540%, respectively, compared to the control sample [133].

It is a known fact that, after cracking, the inclusion of short fibers as a mass reinforcement in fiber-reinforced concrete (FRC) mainly provides crack control due to the tensile stress transfer capability across the crack surfaces of the fibers known as crack bridging. It also improves the energy absorption capacity of the composites structures [134]. In this manner, fibers provide significant resistance to shear across developing cracks and, therefore, FRC demonstrates a pseudo-ductile response, increased residual strength (especially in tension) and enhanced energy dissipations capacities, relative to the brittle behavior of plain concrete mixtures. Furthermore, the advantageous characteristics of FRC under tension are also very important for the shear response of concrete structural members that are governed by the tensile response of the fibrous material. Thus, fibers have proved to be a promising non-conventional reinforcement in concrete elements under shear stresses due to the beneficial cracking performance of FRC, and under specific circumstances, could alter the brittle shear failures to ductile flexural ones. Different researchers worked on the shear critical analysis and reported findings that are useful on the state of the practice and for real-scale constructions [135]. Kytinou et al. reported the flextural properties and enhanced short-term behavior of steel-fiber-reinforced composites through the finite element method. The results explain that type of fibers, their volume fraction and aspect ratio play a significant role in achieving better compressive and tensile properties. Samples with higher amounts of reinforced fibers showed lower deformation at the same applied load than with the lower amount and vice versa. Moreover, a higher amount of steel fibers in reinforced composites show higher post cracking stress and vice versa [136]. Choi et al. also reported the crack mechanism of high-performance FRC that ultimately explain the strain hardening response based on the length of fibers. The results show that tensile properties were significantly overestimated for FRC when individual segments were estimated during a comparison of probabilistic and experimental results. This indicates that fiber tension should not be applied directly in order to abstain from multiple cracks and strain hardening behavior [137].

The study of flowability is another important topic to discuss here. In an experimental study, flowability of the cement paste with three different fibers, polypropylene, polyvinyl alcohol and sisal, was examined. Results revealed that the increase in length and dosage of each fiber decreased the flowability of cement paste. It was further revealed that the flowability of cement paste with sisal fiber was the lowest [138]. Recently, the behavior of sisal-reinforced concrete in exterior beam–column joints under monotonic loads was studied. The authors replaced cement in the concrete mix by 0.5, 1, 1.5 and 2% sisal fibers. Under gradually increasing loads, exterior beam–column joints with sisal-reinforced concrete showed lower deflection and enhanced shear strength [139]. Silva et al. studied the potential use of long aligned sisal fibers in thin cement-based laminates for semi-structural and structural applications. Samples were prepared replacing OPC with 30% metakaolin and 20% calcined waste crushed clay bricks. They prepared calcium-hydroxide-free samples by replacing 50% cement by the calcined clays at 28 days of age. They observed the ultimate tensile strength of calcium hydroxide free composites to be 13.95 MPa with an increase of around 34% and toughness under tensile loads twice that of OPC composites. They further noticed that quicker aging of calcium-hydroxide-free composites through hot water immersion revealed an ultimate strength 3.8 times higher and toughness 42.4 times greater than OPC composites under the same conditions [140].

#### 3.1.2. Jute and Its Composites

**Figure 14:**
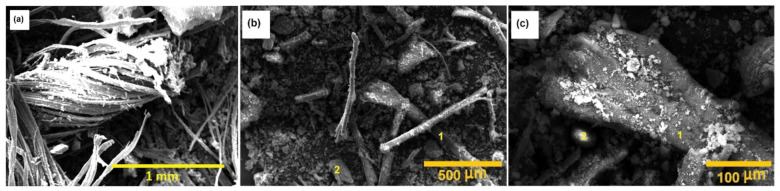
SEM micrographs of (**a**) jute fibers, (**b**) jute-fiber-reinforced geopolymers composites (lower magnification) where 1 shows jute fibril and 2 shows geopolymer aggregates and (**c**) jute fibers reinforced geopolymers composites (higher magnification) where 1 shows jute fibril and 2 shows geopolymer aggregates. Reprinted with permission from Ref. [147]. Copyright 2021, with permission from Frontiersin.

Jute is one of the natural organic cellulosic fibers finding application in green composites and is the most used cellulosic fiber after cotton [141,142,143,144]. Jute is a type of bast fiber obtained from two species, namely Corchorus capsularis and Corchorus olitorius, and is mainly grown in tropical regions of the world including India, Bangladesh and China. Currently, jute production around the world is estimated as 2300 × 10^3^ tons per year. Jute fiber represents a complex mixture of chemical compounds that are produced during the growth of fiber in the plant stem by photosynthesis process. Soil condition, climate, development of the plant and retting process greatly influence the constituents of fiber. In general, jute fiber is made up of 60% cellulose, 22% hemicellulose, 12% lignin, 1% fatty and waxy matter, 1% nitrogenous matter, 1% mineral matter and 3% miscellaneous. The main constituents i.e., cellulose, hemicellulose and lignin, essentially have influence on the fiber’s structure, as others are very minor in proportion [145]. Jute fiber has a relatively low density and high strength and stiffness. In general, jute fiber has a density of around 1.3 g/cm^3^, tensile strength of 393–773 MPa, Young’s modulus of 26.5 GPa and 1.5–1.8% elongation. Jute fiber at 65% relative humidity and at 21 °C has a value of 12 for equilibrium moisture content [146]. Jute is among the most-studied fibers for reinforcement in thermoset and thermoplastic polymers. Alshaaer worked with the synthesis and recyclability of jute-reinforced geopolymers composites and reported four times higher flexural strength of jute-reinforced geopolymers composites as compared to non-reinforced geopolymers composites. Their results are illustrated in Figure 14 [147].

Khondker et al. studied the effects of molding temperature and pressure on mechanical and interfacial properties of polylactic acid and homo-polypropylene-based thermoplastic composites using untreated and treated unidirectional jute yarns. Their results indicated that molding condition at 175 °C and 2.7 MPa pressure was more appropriate for optimized properties of the unidirectional jute fiber/polylactic acid composites. They noticed that in the case of jute fiber/polylactic acid microbraid composites, maximum tensile stress and modulus increased with an increasing fiber volume fraction. In case of jute/homo-polypropylene composites, jute reinforcement caused increase tensile and bending properties of the composites, and with a 20% jute-fiber inclusion, jute/homo-polypropylene composites showed remarkable improvement in tensile and bending properties [148]. Ramakrishnan et al. prepared jute/nano-clay/epoxy hybrid composites employing various ratios of untreated and treated jute fiber and nano-clay and used the compression molding technique to evaluate the dynamic mechanical and free vibration behaviors. They used various NaOH concentrations of 2.5%, 5% and 7.5% to modify the jute fiber surface. In order to improve the dynamic properties of jute-fiber-reinforced epoxy composites, nano-clay 1, 3, 5 and 7 wt.% was added, along with the primary 5% NaOH treated jute fibers. Their research revealed that the dynamic mechanical behavior is greatly influenced by NaOH solution concentration and nano-clay content. Their results indicated that the jute treated with 5% NaOH epoxy composites showed the peak storage modulus value of 3884.56 MPa with a peak loss modulus value of 484.07 MPa. On the other hand, epoxy composites with jute treated with 5% NaOH and 5 wt.% nano-clay showed a storage modulus peak value of 4446.38 MPa and a loss modulus peak value of 544.04 MPa [149]. Yao et al. studied the impact of jute fiber and polyvinyl alcohol (PVA) on flexural and fracture performance of soil-cement column. Their results showed that presence of fibers considerably enhanced the flexural performance and fracture energy of soil-cement. Furthermore, the inclusion of fibers also successfully controlled the formation and propagation of plastic shrinkage cracks at an early age and reduced the flexural strength loss under wet–dry cycles [150].

In a recent study, four fibers, namely piassava, tucum palm, razor grass and jute with 1.5, 3 and 4.5% mass addition of the composite binder, were used as reinforcement in cement mortars to study the mechanical properties with 50% OPC with 40% metakaolin and 10% fly ash. Fibers were treated using four techniques, namely washing in hot water, hornification, 8% sodium hydroxide treatment and hybridization in order to obtain improved physical and mechanical properties. In this respect, sodium hydroxide treatment was selected for jute fiber. Their results showed that treated fibers increased the performance of the composites during mechanical testing. It was further noticed that the inclusion of treated fibers above 3% increased the flexural strength than those without fibers [151]. A study on the mechanical properties of the jute-reinforced geopolymer composite was carried out by Sankar and Kriven. They revealed that the jute-reinforced geopolymer composite showed a flexural strength of 20.5 MPa with an improvement in tensile strength from 8.8 MPa to 14.5 MPa with alkali-treated jute weaves. It was also revealed that the jute-reinforced geopolymer composite could absorb an impact energy of 9.64 J on average [152]. Trindade et al. conducted a study on the mechanical properties of natural fibers using metakaolin as an aluminosilicate source for a geopolymer with sodium hydroxide and sodium silicate as an alkaline activator. A total volumetric fraction of 10% for the Jute and sisal fibers were used for reinforcing the composite, and five layers each of jute and sisal fibers were embedded. Jute- and sisal-fiber-reinforced geopolymer composites showed a significant compressive strength at an average of 72.70 MPa at 7 days. They observed an ultimate tensile strength of 6.31 MPa and an ultimate flexural strength of 15.21 MPa whereas the modulus of elasticity was recorded at 14.92 GPa. They revealed that the jute-fiber-reinforced geopolymer composite exhibited a strain capacity of 28.34 mm [153].

Bheel et al. studied the effect of jute fiber and wheat straw ash on the mechanical properties of concrete. They used 0.25, 0.5, 0.75 and 1% jute fiber as reinforcement and 20, 30 and 40% wheat straw ash as a replacement for fine aggregates. Their results revealed that composites reinforced with 0.5% jute fiber along with 30% wheat straw ash at 28 days were enhanced with values of 32.88 MPa, 3.8 MPa and 5.3 MPa for compressive, splitting tensile and flexural strengths, respectively. Similarly, the modulus of elasticity for all compositions increased as well, reaching 30.45 GPa for composites with 1% jute fiber reinforcement at 28 days. Authors also observed a decrease in permeability and workability of concrete with increasing values of jute fiber and wheat straw ash in concrete [154]. Fonseca et al. prepared fiber-cement-reinforced composites with NaOH-treated jute fibers, cellulose nanofibrils and a hybrid of both Jute fibers and cellulose nanofibrils at 0.5 and 2% mass of cement to study the physical and mechanical properties using an extrusion process. The samples were subjected to natural weathering for 5 months before they were analyzed. Their results revealed that composites reinforced with cellulose nanofabrils, the hybrid (0.5% jute fiber and 1.5% cellulose nanofibrils) and 0.5% jute fiber had the highest apparent density values. With an increase in the proportion of jute fibers and cellulose nanofabrils from 0.5 to 2%, composites showed a reduction in apparent porosity values. The sample reinforced with 2% cellulose nanofabrils showed a 75% reduction in apparent porosity. The composite reinforced with 1.5% cellulose nanofabrils and 0.5% jute fibers showed the strongest mechanical properties. Composites with all compositions showed a reduction in the modulus of elasticity after natural weathering. On the other hand, hybrid reinforced composites showed an increase, on average, of 1 MPa for the modulus of rupture and limit of proportionality [155]. Table 3 summarizes the chemical composition and mechanical properties of sisal and jute fibers.

### 3.2. Inorganic Fiber-Reinforced Geopolymer Composites

The use of inorganic fibers like asbestos dates back to prehistoric time. During the past decades, efforts have been focused on developing high-performance materials that could meet the requirements of improved tensile strength and modulus values. Among them, inorganic fibers with improved mechanical properties have been employed in polymers to produce composites of enhanced properties. Glass, basalt, boron, boron carbide, boron nitride, zirconia, silicon carbide and silicon nitride are among the many inorganic fibers being used for producing composites. Due to their low cost, inorganic fibers are being replaced with carbon fibers in high temperature-resistance applications. The addition of inorganic fibers in polymers significantly improves physical, structural, thermal and rheological behaviors of the composites. Inorganic fibers contain mainly alumina and silica and have high melting temperatures. Low cost, chemical stability and good insulating properties along with enhanced mechanical properties make them attractive for use in different industries including civil engineering. The percentage increase of silica to alumina enhances the tensile strength but it negatively affects the modulus with 100% alumina content. Inorganic fibers with 52% Al_2_O_3_ can withstand 1250 °C, and with a further increase in alumina content, can resist even higher temperatures. Metakaolin-based geopolymers reinforced with high alumina fibers at 600–1000 °C show improved mechanical strength, high energy absorption and reduced shrinkage [156]. However, Welter et al. indicated the weaknesses of some inorganic fiber composites in high-temperature applications [157].

#### 3.2.1. Basalt and Its Composites

**Figure 15:**
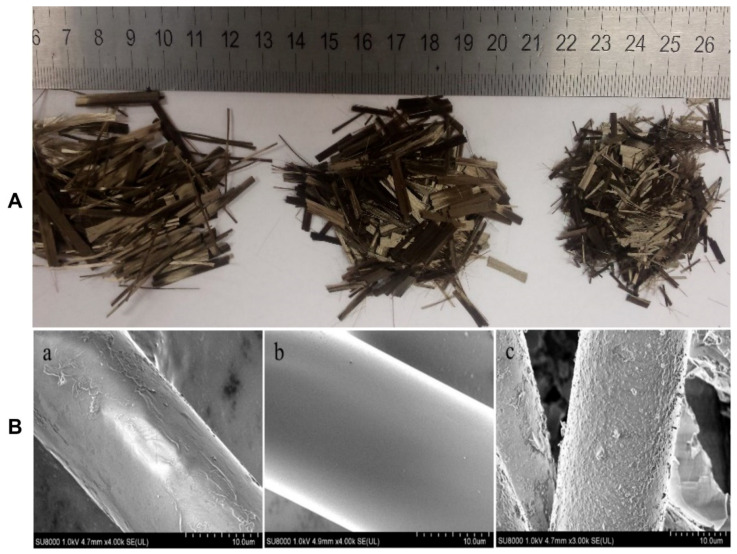
(**A**) Different types of chopped basalt fibers from left to right: With fiber length of 24 mm, 12 mm and 6 mm, respectively, (**B**) morphologies of basalt fibers (**a**) untreated, (**b**) HCl treated and (**c**) NaOH treated. Reprinted with permission from Refs. [165,166]. Copyright 2021, with permission from MDPI and Elsevier.

Basalt, being an eco-friendly, nontoxic and affordable material, is gaining popularity for reinforcing composites. Basalt fiber, among the new materials of the 21st century, is broadly employed in many fields including aerospace, construction, the chemical industry, agriculture, medicine and electronics. Basalt fiber was extensively used in military and aeronautical applications during the second world war. Basalt is obtained from volcanic rocks that are dark in color and form as a result of the solidification of volcanic lava. Basalt flows mainly contain SiO_2_ along with Al_2_O_3_, Fe_2_O_3_, FeO, CaO and MgO. Basalt rocks are classified into alkaline, mildy acidic and acidic according to the SiO_2_ % content present; of which acidic basalts satisfy the conditions for fiber preparation [158]. Basalt fibers are obtained in a fine-fiber shape of 9–13 µm diameter by melting and beating through the centrifugal blowing process of basalt rocks above 1500 °C [159]. Basalt fibers are receiving more attention for use in civil engineering because of their low density, high fatigue strength, low water absorption, good heat and insulation properties, good processability along with cheap fabrication process compared to glass and carbon fibers and high chemical resistance [160,161,162]. Basalt fibers have higher strength values than other inorganic fibers; for example, basalt fibers have a higher tensile strength than E-glass and their strain to failure is greater than that of carbon fiber. Another advantage of basalt fiber is its reasonable resistance to acid attack; however, basalt corrodes in an alkaline environment [163]. Basalt fibers have a high melting point ~1000 °C and are more suitable for high-temperature-resistant applications than cellulosic fibers. Basalt fibers have moisture absorption less than 0.02% for 24 h with a moisture regain value of 1. Basalt fibers show a significant resistance at high temperatures for a short time period, up to 750 °C, and for longer exposure in 260–700 °C and even in some cases up to 1000 °C. Basalt fibers lose 20–25% of their initial strength without losing insulation properties [164]. Chopped basalt fibers with different length and the effect of alkali or acid treatment are very important factors during composite preparation. Acid or base treatments are obliged to add abundant functional groups on the surface of basalt fibers. It is reported that during alkali treatment, basalt fibers provides more rough surface than untreated samples, whereas a very smooth and clean surface is observed for acid treatment as shown in Figure 15 [165].

The inclusion of basalt fibers into carbon-epoxy composites increases the absorbed impact energy of the composites. In a previous study, vinylester and epoxy reinforced with basalt fibers were tested for structural applications. The results showed that the ultimate tensile strength of basalt/epoxy composites increased by 29% and compressive strength increased by 85% as compared to basalt/vinylester composites. It was observed that the failure mode in compression was the same for both types of composites. Kim et al. investigated the effects of modified carbon nanotube/epoxy/basalt on the flexural and fracture properties. Carbon nanotubes were modified with silane treatment and acid treatment. Their results showed that silane-treated carbon nanotube/epoxy/basalt composites had an increase in flexural strength and flexural modulus by 14% and 10%, respectively, compared to acid-treated composites. The fracture toughness of silane-treated composites showed an increase of 40% compared to acid-treated carbon nanotube/epoxy/basalt composites [167]. Szabo and Czigany studied the static properties of short fiber-reinforced polypropylene composites using basalt and ceramic as a reinforcement with 5, 15 and 25 wt.%. Their results showed that fracture toughness greatly depends on the type and direction of the load and thickness. It had been noticed that the characteristic damage form was pull out in transverse and debonding in longitudinal directions [168]. Zhang et al. studied the tensile, flexural and impact properties of basalt-fiber-reinforced poly(butylene succinate) composites. Their results revealed that tensile strength and modulus gradually increased with increasing basalt fiber content. It was observed that tensile strength of the composite increased from 31 MPa to 46 MPa with basalt content increase from 3 vol.% to 15 vol.%. Moreover, the increase in tensile strength at higher loadings of basalt fiber was comparatively smaller than those at lower loadings. Flexural strength of the composite was observed to increase from 18 MPa to 71 MPa with an increase of basalt fiber from 0 to 15 vol.%. Similarly, the flexural modulus also increased from 551 MPa to 3.8 GPa. It was also observed that the impact strength of the basalt-reinforced poly(butylene succinate) did not change at 3 vol.% fiber loading, and with further loading, impact strength increased linearly between 5 and 15 vol.% with its highest value of 7.5 kJ/m^2^ at 15 vol.% [169].

**Figure 16:**
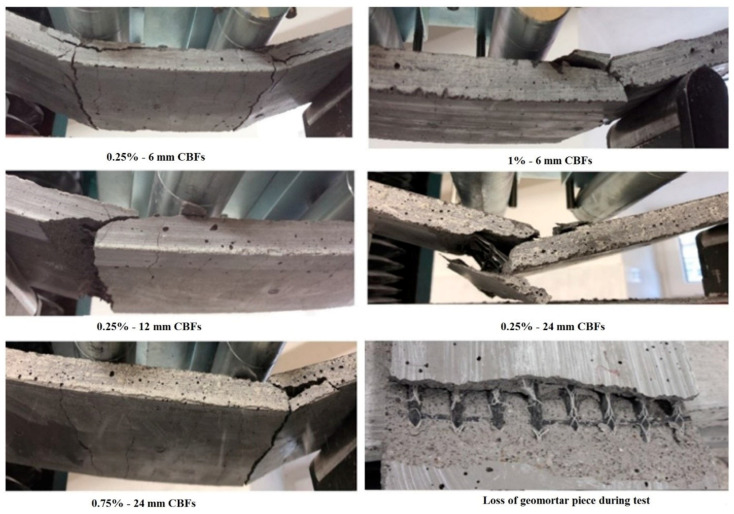
Four-point flexural strength test with varying fiber length of basalt chopped fibers. Reprinted with permission from Ref. [166]. Copyright 2021, with permission from MDPI.

Punurai et al. studied the mechanical properties, microstructure and drying shrinkage of hybrid fly-ash–basalt fiber-reinforced geopolymer paste by replacing fly ash with basalt fiber at 0, 10, 20, 30, 40 and 100%. Their results showed that the inclusion of basalt fiber resulted in increased setting times and the initial, and final setting times with 100% basalt fiber inclusion were 280% and 110% higher than that of 100% fly ash geopolymer paste. The 7-day compressive strength of the geopolymer paste with 40% basalt fiber inclusion was recorded as 112% higher than that of the 100% fly ash geopolymer paste, whereas the 28-day compressive strength of the same sample was 118% higher than the 100% fly ash geopolymer paste. The flexural strength also increased with an increasing content of basalt fiber. The 28-day flexural strength of the sample with 40% basalt fiber content was noted as 64% higher than the 100% fly ash geopolymer paste. Their results indicated that the drying shrinkage of geopolymer paste decreased with increasing basalt fiber content [170]. Figure 16 shows the four-point flexural test results for basalt-fiber-reinforced geopolymers composites [166].

Li and Xu studied the dynamic compressive strength, deformation and energy absorption of basalt-fiber-reinforced geopolymeric concrete. They observed that the inclusion of 0.1 and 0.2% of basalt fiber resulted in 10.1 and 30.9% reduction in dynamic compressive strength whereas with 0.3% basalt fiber, there was no substantial change in dynamic compressive strength. With 0.3% basalt fiber addition to geopolymer concrete, critical strain at the strain rate of 100 s^−1^ increased by 7.7% resulting in improved deformation capacity of the geopolymer concrete. Furthermore, the addition of 0.3% basalt fiber results in an increase of 8.9–13.2% in specific energy absorption at the strain rate from 40 to 100 s^−1^ showing a noticeable improvement in energy absorption capacity [171]. Yang et al. studied the effects of basalt fiber content on the uniaxial compressive mechanical properties of concrete. Their research showed that 6 kg/m^3^ of basalt fiber in concrete could improve the compressive strength and could reduce the density and intensity of acoustic emission. They concluded that with the increase in basalt fiber content, local damage could be effectively weakened. Their results showed that the proper amount of basalt fiber in concrete delayed the early cracking and reduced the transverse strain of concrete [172]. Shen et al. conducted a study on reinforced concrete beam-column joints by strengthening them with basalt-fiber-reinforced polymer sheets in different ways under cyclic loads. Their results showed that overall seismic performance of the joints strengthened with basalt-fiber-reinforced polymer sheets was enhanced noticeably, and there was good interface behavior between the concrete and basalt-reinforced polymer sheets. They also observed that load-bearing capacity, ductility and stiffness of joints increased by strengthening with basalt-fiber-reinforced polymer sheets. Likewise, the energy dissipation capacity of the joints with basalt-fiber-reinforced polymer sheets also increased [173].

#### 3.2.2. Glass and Its Composites

**Figure 17:**
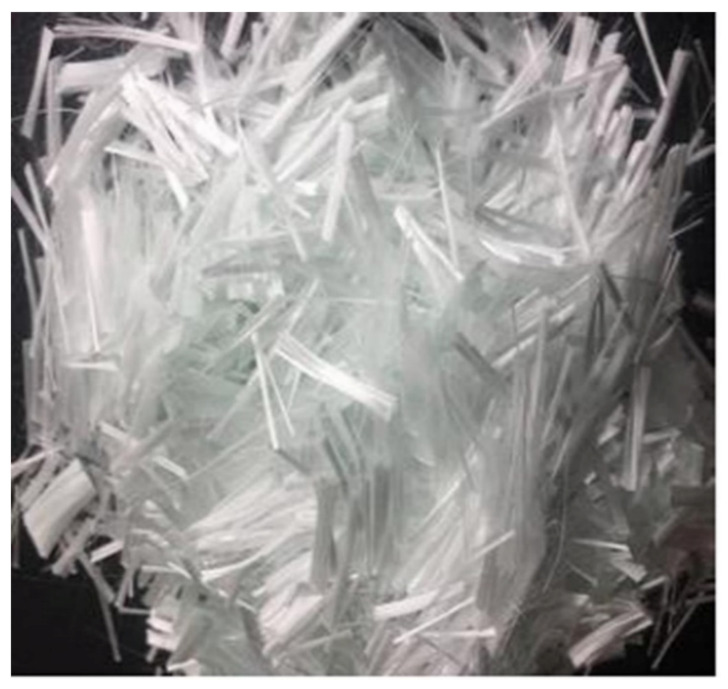
AR glass fibers. Reprinted with permission from Ref. [180]. Copyright 2021, with permission from Elsevier.

Glass fiber is one of the most common reinforcement in polymer composites. It is a strong, less brittle, lightweight and cost-effective material. The application areas of glass fibers include automobile, marine, sports, leisure goods, aerospace and the construction industry [174,175,176]. In the construction industry, glass fibers are mainly used for the production of fibrocement-based objects and for external strengthening of existing buildings. The global production of glass fiber accounts for about 5 million metric tons annually and it is estimated that the global market value will reach more than $21 billion in 2025. The use of glass dates back to ancient times e.g., many ancient Egyptian ships were made by winding glass fibers on a rim of clay of appropriate form. Commercial production of glass fiber started in 1930s by the Owens-Illinois Glass Company. There exist many groups of glasses including silica, oxynitride, phosphate and halide of which silica glasses are used for composites reinforcement. Glass fibers are manufactured by using different types of broken glass that contain silica along with other components like alumina, oxides of calcium, magnesium and boron. The manufacturing process of glass fiber involves high-temperature conversion of raw materials into a homogeneous melt that is later converted into glass fibers. The production process has three phases, namely raw material handling, glass melting and refining and fiber formation. Glass fibers are produced in many forms, including continuous fiber, rovings, staple fiber and chopped strand. The mixing of continuous and chopped-strand glass fibers with resin is more common. Depending on the chemical composition and end use, glass fibers are characterized into many classes, namely C-glass, D-glass, R-glass, E-glass and S-glass [177]. E-glass, S-glass and C-glass are the leading glass fibers, with E-glass being most widely used in composites because of its low cost and relatively low moduli. S-glass fibers are stiffer and stronger than E-glass and have better resistance to fatigue and creep. E-glass are alumino-borosilicate and are mainly used for glass-reinforced plastics, while S-glass are also alumino-silicate with no CaO content and the highest value of tensile strength among all glass fibers and are mainly used in aircraft components and missile casings [178]. AR-glass fibers represent good resistance in alkaline media and are being used in cement substrates and concrete. C-glass fibers have good resistance to chemical resistance [179]. Figure 17 gives a random orientation of AR-glass fibers [180].

The main advantages of glass fibers are excellent high tensile strength and low cost of production. The structure of glass fibers is amorphous and the Young’s modulus of glass fiber is same as in the bulk form of glass. While the strength-to-weight ratio of glass fibers is high and elastic modulus is low, they increase stiffness and reduce elongation of plastic composites. Glass fibers have a high ratio of surface area to weight, and this makes them vulnerable to chemical attacking. On the other hand, glass fibers have a good thermal insulation property. The main drawbacks of glass fibers are their relatively low elasticity modulus, reduced long-term strength and weak resistance to moisture and alkaline mediums. Other drawbacks of glass fibers are their high sensitivity to abrasion during handling, poor fatigue resistance and high hardness.

Faizal et al. studied the tensile behavior of a plane-woven E-glass fiber-reinforced polyester composite. Composites were prepared using a symmetrical and non-symmetrical lay-up of glass fibers and were cured at different curing pressures. Their results revealed that for both symmetrical and non-symmetrical lay-ups, the tensile modulus decreased with increasing curing pressure. They observed a common stiffness characteristic for both symmetrical and non-symmetrical arrangements at a curing pressure of about 87.1 kg/m^2^. For the symmetrical lay-up arrangement, ductility decreased with an increasing curing pressure whereas it increased with increasing curing pressure in the case of the non-symmetrical lay-up arrangement [181]. Kushwaha and Kumar investigated the mechanical properties of the bamboo glass mat (strand and woven)-reinforced epoxy and polyester laminate composites. They found that in the glass strand mat and bamboo epoxy composites, improved tensile strength and tensile modulus could be achieved with a comparatively lower weight percentage of glass fibers along with bamboo fiber reinforcement. The same behavior was noticed for flexural strength as well; however, the flexural modulus increased with higher percentages of glass fiber, whereas in woven glass mat and bamboo-reinforced epoxy composites, tensile strength and modulus and flexural strength and modulus increased with the increase in glass fiber content. The same results were observed for the polyester-based composites [182]. Devendra and Rangaswamy investigated the mechanical properties of E-glass fiber-reinforced epoxy composites using varying concentrations of fly ash, aluminum oxide, magnesium hydroxide and hematite powder as fillers. Their results revealed that the composite filled by 10 vol.% of magnesium hydroxides showed the maximum ultimate strength when compared with other filled composites. High impact strength was observed with 10 vol.% of fly ash, whereas aluminum oxide and magnesium hydroxide showed good impact strength at 10 vol.% and their further increase led to a reduction in impact strength. Results showed that impact strength increased with the increasing amount of hematite powder. Furthermore, the hardness of composites increased with an increasing amount of magnesium hydroxide, aluminum oxide and hematite and decreased with the increasing amount of fly ash. Magnesium hydroxide exhibited the highest hardness number when compared to other fillers [183].

Etcheverry and Barbosa studied the glass fiber/polypropylene adhesion improvements. They revealed that in-situ metallocenic polymerization of propylene on the glass fiber surface increased the adhesion between polypropylene matrix and glass fiber reinforcement [184]. In a previous study, the mechanical behavior of E-chopped strand glass fiber-reinforced wood sawdust/polyvinyl chloride composites with 50% wood were examined. The varying percentages of glass fiber were 10, 20 and 30% with initial fiber lengths of 3, 6 and 12 mm. The results showed that the stiffness and strength of wood sawdust/polyvinyl chloride composites improved with increasing glass fiber content. Composites reinforced with 30% glass fiber with a final length showed greater tensile and flexural strength and moduli. Increasing the glass fiber content led to an increased impact strength of wood/polyvinyl chloride composites, whereas elongation at break slightly decreased with an increasing content of glass fiber. A reduction in percentage shrinkage of the composites occurred with increasing glass fiber content [185]. Chen et al. studied the mechanical properties of a polyamide66/polyphenylene sulphide blend reinforced with 5, 10, 20 and 30% volume content of glass fiber. Their results showed that the inclusion of glass fiber significantly improved the tensile strength, flexural strength and hardness of the composites but decreased the impact strength of the blend. The maximum tensile strength and flexural strength were achieved with 30 vol.% and with 25 vol.% of glass fiber, respectively [186]. Cheng et al. investigated the mechanical properties of glass-fiber-reinforced cement with fly ash or slag after natural curing for 28, 180 and 360 days and accelerated aging at 80 °C for 8 days. They used 3% glass fiber content by weight of the total solid mix by replacing an equal quantity of sand, whereas fly ash or slag were added at a ratio of 0%, 20% and 40%, respectively. Their results showed that regardless of admixture, the modulus of rupture of glass-reinforced cement increased with an increase in curing time and its value was noted as being higher than that of the mortar without glass fiber. They further observed that after 8 days of accelerated aging at 80 °C, the modulus of rupture of glass-reinforced cement without any admixture decreased radically and had a lower value than that of glass-reinforced cement with natural curing for 360 days and mortar without glass-fiber accelerated curing for 880 °C. They concluded that fly ash and slag could improve the long-term strength of glass-reinforced cement but could not constrain the toughness degradation of glass reinforced cement mortars [187]. Fang et al. investigated the compressive, flexural strength and water resistance of fiber-glass-reinforced magnesium phosphate cement mortar with fiber volume fractions of 1.5%, 2.5%, 3% and 3.5%, respectively. Their results revealed the optimal volume fraction of glass fiber at 2.5% and that the glass fibers had more noticeable effects on the flexural strength than on compressive strength. They further observed that the water-resistance performance in the compressive and flexural strength might not be improved with glass fiber magnesium phosphate cement mortars [188]. Gese et al. investigated the performance of AR-glass-reinforced mortar composite in flexural strengthening of RC beams considering three factors, namely age (3, 7 and 28 days) of AR-glass reinforced mortar, number (2, 3 and 4) of AR-glass-reinforced mortar layers and the pre-cracking level (no pre-cracking, 50% and 100% of the yielding load). It was observed that the AR-glass-reinforced mortar external reinforcement decreased the ductility of the beams. Their results showed a 49% increase in yield load for 28-day beams and 33–30% for 3–7-day beams, respectively. The ultimate load was considerably improved by AR-glass reinforcement and increased by 31%, 54% and 72% for the beams strengthened with two, three and four layers, respectively, whereas AR-glass-reinforced mortar ages and pre-cracking had no significant differences in the ultimate load for beams. Although pre-cracking affected the crack load, it modified the behavior of stage II of the flexural test. Higher stiffness noted in stage II implied better performance in pre-cracked beams for yield loads than that of crack load increase [189]. Table 4 summarizes the chemical composition and the mechanical properties of basalt and different types of glass fibers.

## 4. Summary and Future Direction

- This paper represents the inclusion of selective cellulosic and non-cellulosic fibers in geopolymers-based, fiber-reinforced concrete composites from a construction and civil engineering perspective. Geopolymers are the relatively new materials being employed in the construction industry to replace the use of traditional concrete materials. Due to a number of advantages, interest in developing, characterizing and implementing the use of geopolymers in the construction industry is growing.
- Geopolymer cement-based materials are developed using alumina silicate sources, with fly ash, metakaolin and GGBFS being the most-used ones. First, the properties and uses of these alumino-silicate materials were briefly discussed and represented in this paper. Moreover, the second part discussed the inclusion of fibers as a reinforcement in concrete composites. It is well-known that geopolymers alone cannot respond adequately to certain mechanical properties and hence need to be employed in combination with other suitable materials.
- As discussed in this paper, geopolymers are weak in tension and possess brittle behavior that represents poor tensile/flexural properties. To overcome this problem, one such solution is the inclusion of fibers in geopolymers-based composites. Natural fibers are gaining attention regarding their use in composites due to a number of reasons, including their relatively low density, excellent strength and environmental friendliness.
- This paper examines the use of cellulosic and non-cellulosic fibers in composites for the construction industry. A brief description of sisal, jute, basalt and glass fibers are discussed in this context and represent some recent works conducted in the area.

## Figures and Tables

**Table 1:** A brief summary of the constituents of geopolymers.

Constituents	Composition	Temperature [°C]	Time [Days]	Chemicals	Compressive Strength [MPa]	References
Coal fly ash & metakaolin	-	250	3	NaOH	-	[34]
Fly ash, GGBFS & zeolite	Al/Si	32	1	NaOH	100	[35]
Coal fly ash	Al/Si	80	1	NaOH	18	[36]
Fly ash	SiO_2_/Al_2_O_3_	85	3	KOH	19	[37]

**Table 2:** Chemical composition and physical properties of cementitious materials used in geopolymers concrete composites.

**Fly Ash**	**Coal Type**	**Chemical Composition (wt.%)**									**Physical Properties**			**Ref.**
		**SiO_2_**	**CaO**	**Al_2_O_3_**	**MgO**	**SO_3_**	**Fe_2_O_3_**	**K_2_O**	**Na_2_O**	**TiO_2_**	**Specific Surface Area (m^2^/kg)**	**Specific Gravity**	**Bulk Density (kg/m^3^)**	
	Sub-bituminous	40–60	5–30	20–30	1–6	0–2	4–10	0–4	0–2	-	170–1000	2.1–3.0	540–860	[61,62]
	Lignite	15–45	15–40	10–25	3–10	0–10	4–15	0–4	0–6	-				
	Bituminous	20–60	1–12	5–35	0–5	0–4	10–40	0–3	0–4	-				
	Anthracite	28–57	1–27	18–36	1–4	0–9	3–16	0–4	0–1	-				
GGBFS		28–40	30–50	8–24	1–18	0.23–1.3	-	-	-	-	300–500	2.4–3.0	1200	[73,75]
Metakaolin		51.9	0.11	45.39	-	-	0.92	0.45	-	0.76	10,000–29,000	2.2–2.6	300–400	[82,85,86]

**Table 3:** Chemical composition and properties of cellulosic fibers.

Fiber Name	Chemical Composition (wt.%)			Physico-Mechanical Properties					Ref.
	Cellulose	Hemicellulose	Lignin	Density (g/cm^3^)	Tensile Strength (MPa)	Young’s Modulus (GPa)	Elongation at Break (%)	Equilibrium Moisture Content (%)	
Sisal	67–78	10–14.2	8–11	1.03–1.45	347–700	15.4–18.79	2–2.5	12	[119,122,123]
Jute	60	22	12	1.3	393–773	26.5	1.5–1.8	12	[145,146]

**Table 4:** Chemical composition and properties of inorganic fibers.

Fiber Name	Chemical Composition (wt.%)								Physico-Mechanical Properties				Ref.
	SiO_2_	Al_2_O_3_	CaO	MgO	Fe_2_O_3_	Na_2_O	B_2_O_3_	Others	Density (g/cm^3^)	Tensile Strength (MPa)	Young’s Modulus (GPa)	Elongation at Break (%)	
Basalt	52.8	17.5	8.59	4.63	10.3	3.34	-	~3.34	2.65–2.83	3000–4840	89–110	3–3.15	[99,158]
E-glass	52–56	12–16	16–25	0–5	-	-	5–10	-	2.58	1.7–3.5	69–72	4.8	[177]
S-glass	65	25	-	10	-	-	-	-	2.48	2–4.5	85	5.7	
AR-glass	55–75	0–5	1–10	-	-	-	0–8	-	2.7	3.24	73.1	4.4	
C-glass	65	4	14	3	-	-	5.5	-	2.52	1.7–2.8	68.9	4.8	

## Data Availability

Not applicable.
